# Longitudinal Femoral Cartilage T2 Relaxation Time and Thickness Changes with Fast Sequential Radiographic Progression of Medial Knee Osteoarthritis—Data from the Osteoarthritis Initiative (OAI)

**DOI:** 10.3390/jcm10061294

**Published:** 2021-03-21

**Authors:** Shannon N. Edd, Patrick Omoumi, Brigitte M. Jolles, Julien Favre

**Affiliations:** 1 Swiss BioMotion Lab, Department of Musculoskeletal Medicine (DAL), Lausanne University Hospital and University of Lausanne (CHUV-UNIL), 1011 Lausanne, Switzerland; shannonedd@alumni.stanford.edu (S.N.E.); brigitte.jolles-haeberli@chuv.ch (B.M.J.); 2 Department of Diagnostic and Interventional Radiology, Lausanne University Hospital and University of Lausanne (CHUV-UNIL), 1011 Lausanne, Switzerland; 3 Ecole Polytechnique Fédérale de Lausanne (EPFL), Institute of Microengineering, 1015 Lausanne, Switzerland

**Keywords:** knee, osteoarthritis, magnetic resonance imaging, cartilage, composition, morphology, three-dimensional, spatial variations

## Abstract

This study tested for longitudinal changes in femoral cartilage T2 relaxation time and thickness in fast-progressing medial femorotibial osteoarthritis (OA). From the Osteoarthritis Initiative (OAI) database, nineteen knees fulfilled the inclusion criteria, which included medial femorotibial OA and sequential progression from Kellgren–Lawrence grade (KL) 1 to KL2 to KL3 within five years. Median T2 value and mean thickness were calculated for six condylar volumes of interest (VOIs; medial/lateral anterior, central, posterior) and six sub-VOIs (medial/lateral anterior external, central, internal). T2 value and thickness changes between severity timepoints were tested using repeated statistics. T2 values increased between KL1 and KL2 and between KL1 and KL3 in the medial compartment (*p* ≤ 0.02), whereas both increases and decreases were observed between the same timepoints in the lateral compartment (*p* ≤ 0.02). Cartilage thickness decreased in VOI/subVOIs of the medial compartment from KL1 to KL2 and KL3 (*p* ≤ 0.014). Cartilage T2 value and thickness changes varied spatially over the femoral condyles. While all T2 changes occurred in the early radiographic stages of OA, thickness changes occurred primarily in the later stages. These data therefore support the use of T2 relaxation time analyses in methods of detecting disease-related change during early OA, a valuable period for therapeutic interventions.

## 1. Introduction

Magnetic resonance imaging (MRI) relaxometry methods allow for in vivo analyses of cartilage tissue properties and detection of cartilage degradation associated with knee osteoarthritis (OA) [1]. Specifically, cartilage T2 relaxation time has been related to tissue water and collagen content, along with the structural organization of cartilage [1]. Cross-sectional studies indicate that T2 relaxation times are longer in OA compared to healthy knees, and they are increased with greater radiographic severity of OA [2]. However, longitudinal studies of T2 changes with disease progression have reported inconsistent results, with indications of both no longitudinal femoral T2 changes [3] and increases in T2 values [4] in knees of various OA severities. These discrepancies are likely due to the various definitions of structural progression, follow-up time periods, OA severity, and regions of interest [3,4]. The application of a standardized, validated method of assessing longitudinal cartilage composition changes could provide increased clarity herein [5]. Furthermore, previous studies have assessed changes in T2 over specified durations of follow-up (e.g., 12 months) [3,4]. However, future T2 analysis applications, particularly regarding the measurement of OA disease progression, require an understanding of the evolution of T2 relaxation times through several OA severity grades. Because disease severity and progression is often assessed using radiographic Kellgren–Lawrence grading (KL), it is especially interesting to study the longitudinal evolution of T2 relaxation time in knees progressing sequentially through various KL stages [6].

Testing for T2 longitudinal changes throughout multiple KL stages requires frequent imaging data acquisition for a cohort of progressing OA knees, which is available through the Osteoarthritis Initiative (OAI) database. Here, MRI and radiographic data was collected for nearly 5000 community members over an eight-year follow-up period. Because OA progression can advance at vastly different rates, it is important to consider the progression speed when conducting analyses of longitudinal cartilage changes [7]. Given the limitations in follow-up period in the OAI, a group of “fast progressing” knees, defined as knees advancing from KL1 to KL3 within 5 years, provides a homogeneous population of knees for cartilage analyses [8]. Additionally, including analyses of KL2 provides a common mid-point in the progression of OA. Therefore, in order to characterize the relationship between sequential radiographic progression of OA and changes in cartilage properties, a subset of OAI knees that progressed sequentially from KL1 to KL2 to KL3 within five years was selected for this study.

The known spatial variations of T2 values support the analysis of changes in relaxation time over the entire cartilage surfaces [5,9]. A recently developed technique allows for quantifying changes in T2 in a set of volumes of interest (VOIs) over the entire femoral condyles that are consistent with cartilage morphology analyses [5]. This method also avoids the difficulties faced with segmenting cartilage on T2 MR images by performing cartilage segmentation on high resolution morphological MR images and combining this data with the relaxometry data [5]. As such, information regarding the spatial variations in both cartilage morphology and composition can be derived from a single image segmentation process. Therefore, this study aimed to employ this previously verified MRI relaxometry and morphology analysis methods to characterize cartilage T2 and thickness changes in medial OA knees progressing sequentially from mild to moderate OA, from KL1 to KL2 to KL3. Based on previous cross-sectional and short-term longitudinal studies [2,4], it was hypothesized that T2 relaxation times would increase monotonically from KL1 to KL2 to KL3 throughout the femoral condyles. From studies of the medial knee OA population, femoral cartilage thickness was hypothesized to increase in the medial load-bearing area from KL1 to KL2, and decrease from KL2 to KL3 [10,11,12,13,14].

## 2. Methods

### 2.1. Study Population

Data used in this study are from the Osteoarthritis Initiative (OAI) public use datasets (https://oai.nih.gov). Specific datasets included 0.2.3 for baseline clinical data along with 0.E.2, 1.E.2, 3.E.2, 5.E.2, 6.E.3, 8.E.2, and 10.E.1 for imaging data (see Appendix A, Table A1 for further information). 

**Figure 1:**
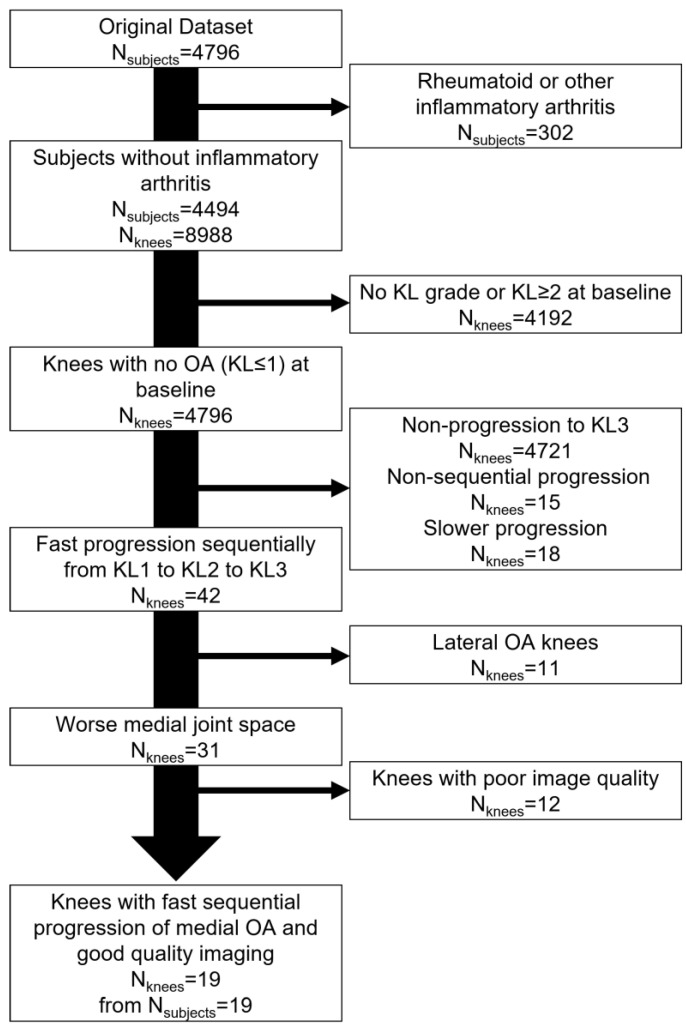
Flowchart of study inclusion.

Study knees that progressed monotonically from KL1 to KL2 to KL3 within 60 months [8] with worse medial (versus lateral) joint space narrowing and satisfactory image quality were included. As depicted in Figure 1, this resulted in a study group of 19 knees from 19 subjects.

### 2.2. Imaging Analysis

MRI images at the first timepoints in which each KL grade was reported (“severity timepoint”) were analyzed as previously described [5]. Briefly, the 3D double echo steady state (DESS) MRI (repetition time (TR) = 16.3 ms; echo time (TE) = 4.7 ms; resolution 0.365 × 0.456 × 0.7 mm) were segmented to create bone and cartilage three-dimensional models from which cartilage thickness maps were computed [15,16]. A single operator (C.D.), blind to all patient data including KL grades, segmented all study knees at the three severity timepoints in random order. The segmentation operator trained with a musculoskeletal radiologist with over ten years of experience (P.O.), and all segmentations were reviewed by the senior radiologist. Using the constructed bone and cartilage three-dimensional models, cartilage thickness was computed at each vertex of the subchondral bone model as the minimum distance to the cartilage model surface. The intra- and inter-operator reliability of femoral CTh has been shown to be excellent with this method (ICC ≥ 0.82) [17]. Mean cartilage thickness was then computed per VOI. 

**Figure A1:**
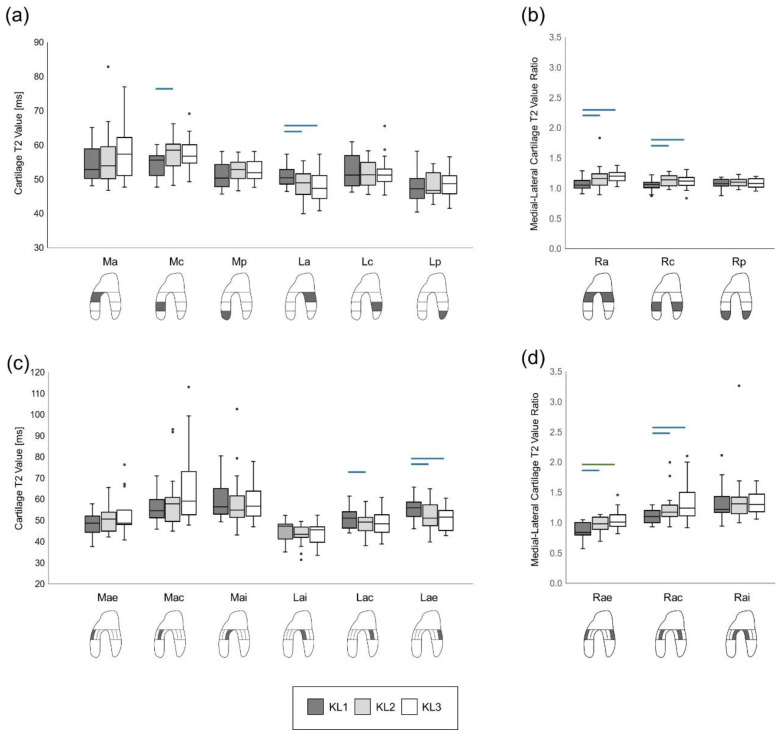
Box and whisker plots of superficial cartilage T2 values per VOI (**a**) and sub-VOI (**c**) and their respective medial-lateral ratios (**b**,**d**). Horizontal bars indicate statistically significant (*p* < 0.017) changes between severity timepoints (blue: ES > 0.5; green: ES > 0.8).

**Figure A2:**
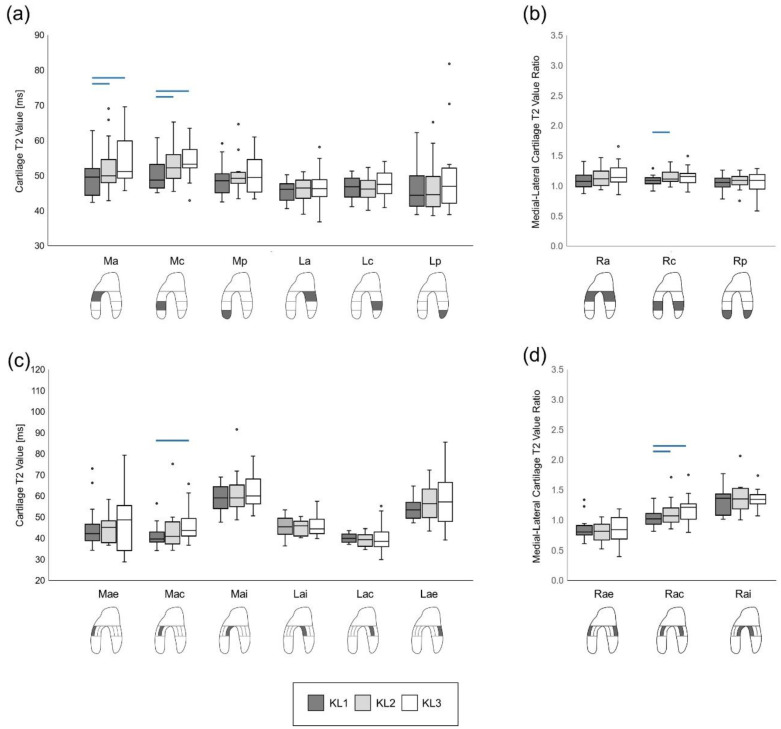
Box and whisker plots of deep cartilage T2 values per VOI (**a**) and sub-VOI (**c**) and their respective medial-lateral ratios (**b**,**d**). Horizontal bars indicate statistically significant (*p* < 0.017) changes between severity timepoints (blue: ES > 0.5; green: ES > 0.8).

The three-dimensional bone and cartilage models were also semi-manually registered to the multi-slice, multi-echo (MSME) MRI T2 sequence (TR = 2700 ms; TE = 10, 20, 30, 40, 50, 60, 70; resolution 0.313 × 0.446 × 3 mm), as previously described [5,18]. Briefly, using custom software, spatial information from the DESS and MSME DICOM headers were used to make an initial registration. The models were then manually translated and rotated to match the tissue structures shown in the MSME images [5]. Intra- and inter-rater reliabilities of this method have been reported as good to excellent (ICC ≥ 0.66) [5]. Per-voxel T2 values were calculated using a monoexponential fit of signal intensities in echoes two through seven, thus excluding the first echo to minimize the effect of stimulated echoes [5,19]. Due to the non-normal distribution of T2 values (as per Shapiro–Wilk tests for normality), median T2 relaxation times were calculated for the full cartilage thickness per (sub-)VOI, excluding values strictly less than 0 ms and values greater than the median plus three times the interquartile range, as per Raya et al. [5,20]. As a supplementary analysis (Appendix B), median T2 relaxation times were also computed in each (sub-)VOI for the superficial (Figure A1) and deep (Figure A2) layers, divided at half cartilage thickness.

**Figure 2:**
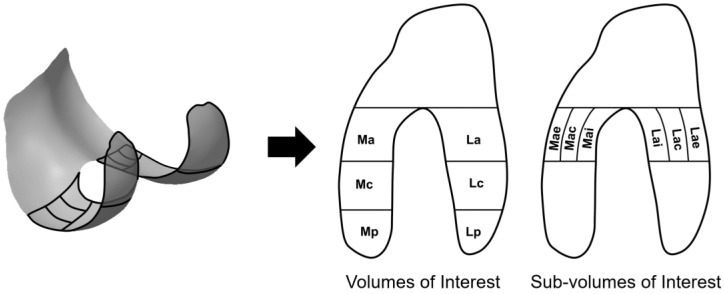
Cartilage volumes of interest (VOIs) and sub-VOIs displayed on a representative femur.

VOIs covered the entire condylar cartilage, dividing it into three parts from anterior to posterior as medial and lateral anterior (Ma, La), central (Mc, Lc), and posterior (Mp, Lp) VOIs (Figure 2). Ma and La were further divided along the medial-lateral axis into medial and lateral anterior external (Mae, Lae), anterior central (Mac, Lac), and anterior internal (Mai, Lai) sub-VOIs. Medial-lateral ratios were also calculated (e.g., Ra = Ma/La, Rae = Mae/Lae), as is commonly done in studies of cartilage thickness. Per convention, both the anterior and central VOIs are considered load-bearing [21].

### 2.3. Statistical Analysis

Non-parametric statistics were utilized as T2 values per (sub-)VOI were found not to be normally distributed across the study knees, as determined using the Shapiro–Wilk test for normality. Therefore, to test for changes in group median T2 relaxation time per (sub-)VOI between severity timepoints, Friedman nonparametric repeated measures tests followed by Wilcoxon signed-rank tests were performed. 

The cartilage thickness data was normally distributed across study knees and thus repeated measures analysis of variance (ANOVA) was used to test for changes in (sub-)VOI mean cartilage thickness between the three severity timepoints; post-hoc paired t-tests were conducted when indicated. 

Alpha was set at 0.05 for the repeated measures test per (sub-)VOI and was Bonferroni-corrected for multiple comparisons for the post-hoc pairwise testing (α = 0.017). Effect sizes (ES) were calculated to quantify the magnitude of the changes in post-hoc comparisons [22].

All statistical calculations were performed using SPSS software (version 23, IBM, New York, NY, USA).

## 3. Results

The 19 participants (8 females and 11 males) had an average (standard deviation) age of 60.7 (7.8) years and BMI of 31.1 (4.6) kg/m^2^ at the KL1 severity timepoint. The median time between KL1 and KL3 was 36 (first quartile: 30, third quartile: 45) months.

### 3.1. Cartilage T2 Analyses

**Figure 3:**
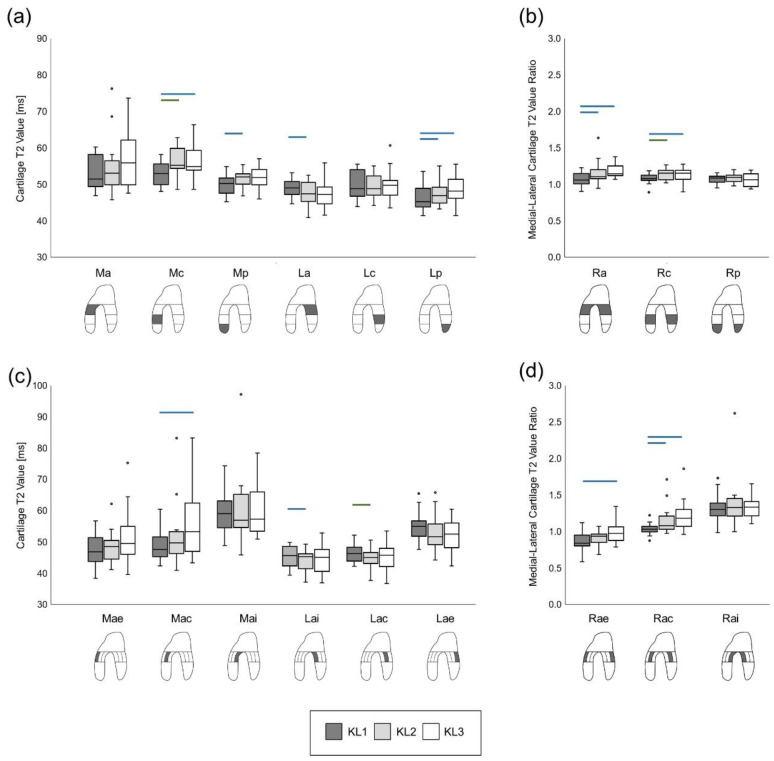
Box and whisker plots of cartilage T2 values per VOI (**a**) and sub-VOI (**c**) and their respective medial-lateral ratios (**b**,**d**). Horizontal bars indicate statistically significant (*p* < 0.017) changes between severity timepoints (blue: ES > 0.5; green: ES > 0.8).

Friedman repeated measures testing indicated significant severity timepoint effects on cartilage T2 values for all VOIs (*p* < 0.02), except Ma (*p* = 0.14) and Lc (*p* = 0.62). Pairwise comparisons indicated that, between KL1 and KL2 severity timepoints, T2 values increased significantly in the Mc, Mp, and Lp VOIs (*p* ≤ 0.02, ES ≥ 0.57), but significantly decreased in the La VOI (*p* = 0.003, ES = 0.68) (Figure 3a). A significant increase in T2 values was also reported between KL1 and KL3 in the Mc and Lp VOIs (*p* < 0.005, ES ≥ 0.65). Furthermore, significant severity timepoint effects were found for all sub-VOIs (*p* < 0.017), except Mae (*p* = 0.08) and Mai (*p* = 0.08). T2 values increased significantly between KL1 and KL3 in the Mac sub-VOI (*p* = 0.005, ES = 0.55), while significant decreases were observed between KL1 and KL2 in the Lac (*p* < 0.001, ES = 0.82) and Lai (*p* = 0.01, ES ≥ 0.59) sub-VOIs (Figure 3c).

Significant severity timepoint effects were observed for the anterior (Ra) and central (Rc) VOI medial-lateral ratios (*p* < 0.001). Here, ratios increased both between KL1 and KL2 (*p* ≤ 0.006, ES ≥ 0.63) and between KL1 and KL3 (*p* ≤ 0.01, ES ≥ 0.59) (Figure 3b). There were significant severity timepoint effects in the anterior external (Rae) and central (Rac) sub-VOI medial-lateral ratios (*p* < 0.008). Rae increased between KL1 and KL3 (*p* = 0.001, ES = 0.77), whereas Rac increased both between KL1 and KL2 (*p* = 0.001, ES ≥ 0.77) and between KL1 and KL3 (*p* = 0.004, ES = 0.64) (Figure 3d).

Results for the supplementary analyses of T2 values in the superficial and deep cartilage layers are provided in Appendix B (Figure A1 and Figure A2).

### 3.2. Cartilage Thickness Analyses

**Figure 4:**
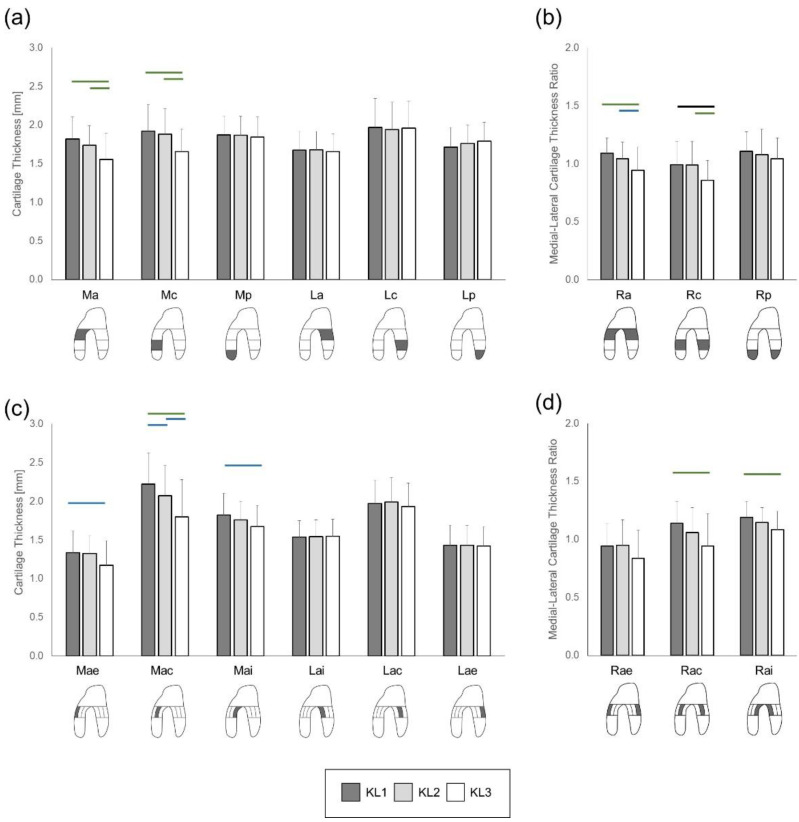
Bar plots (mean ± standard deviation) of cartilage thickness per VOI (**a**) and sub-VOI (**c**) and their respective medial-lateral ratios (**b**,**d**). Horizontal bars indicate statistically significant (*p* < 0.017) changes between severity timepoints (blue: ES > 0.5; green: ES > 0.8; black: ES > 1.2).

The ANOVA reported significant timepoint effects on cartilage thickness in the Ma and Mc VOIs (*p* < 0.001). In these VOIs, the post-hoc comparisons indicated significant decreases between KL1 and KL3 and between KL2 and KL3 (*p* < 0.003, ES ≥ 0.0.81) (Figure 4a). Regarding the sub-VOIs, significant differences among severity timepoints were observed in Mae (*p* = 0.017), Mac (*p* < 0.001), and Mai (*p* = 0.009). Post-hoc comparisons indicated a significant decrease in cartilage thickness from KL1 to KL3 timepoints in these three sub-VOIs (Mae: *p* = 0.014, ES = 0.63; Mac: *p* < 0.001, ES = 1.1; Mai: *p* = 0.004, ES = 0.77) (Figure 4c). Additionally, cartilage thickness significantly decreased in the Mac sub-VOI from KL1 to KL2 (*p* = 0.012, ES = 0.64) and from KL2 to KL3 (*p* = 0.004, ES = 0.76). No severity timepoint effect was detected in the Mp VOI (*p* = 0.5), nor in the lateral VOIs or sub-VOIs (*p* > 0.12).

Significant severity timepoint effects were observed for the anterior (Ra) and central (Rc) VOI medial-lateral ratios (*p* < 0.001), with post-hoc analysis indicating significant decreases between KL1 and KL3 and between KL2 and KL3 for both ratios (*p* < 0.007, ES ≥ 0.7) (Figure 4b). Significant severity timepoint effects were also reported for the anterior central (Rac) and internal (Rai) VOI medial-lateral ratios (*p* < 0.002). The post-hoc analysis indicated significant thickness decreases from KL1 to KL3 in the Rac and Rai ratios (*p* < 0.001, ES ≥ 0.93) (Figure 4d).

## 4. Discussion

The results demonstrated that, in radiographically fast-progressing medial OA knees, T2 relaxation times increased in the medial femoral condyle and in the posterior lateral condyle. Furthermore, contrary to the study hypotheses, decreases in T2 values were found in the anterior area of the lateral compartment. These opposing changes between the medial and lateral compartments were further demonstrated in the medial-lateral ratios, which increased in both the anterior and central areas of cartilage. Interestingly, there were no significant changes in T2 relaxation time at any location between KL2 and KL3 severity timepoints, suggesting that T2 changes occur early in the disease process. This is consistent with previous reports of increased T2 values in at-risk populations [23,24] and the predictive capacity of T2 values for subsequent OA development [25].

Simultaneously, a decrease in cartilage thickness was observed in the medial load-bearing VOIs primarily between KL2 and KL3 radiographic stages, later in the disease than T2 changes. These results agree with the location of cartilage thinning previously observed in the general medial knee OA population, with medial areas of greater loading displaying the greatest loss in cartilage thickness [10,26,27]. In examining the sub-VOIs of the medial anterior VOI, significant thinning was detected first in the medial anterior central sub-VOI from KL1 to KL2 and then in the medial anterior internal and external sub-VOIs. This is also consistent with previous longitudinal [10,12] and cross-sectional data [13,14,28], in which cartilage loss was reported to occur first in the anterior central sub-VOI and then grow toward the exterior.

Taken together, the results suggest that T2 relaxation time changes precede cartilage thickness loss in OA disease progression. Thus, T2 relaxation time analyses could be especially useful for evaluating disease progression during the early development of OA, before the occurrence of any substantial chondral tissue loss. This is a particularly important period in which interventions could be most effective in preserving the existing cartilage tissue. 

The observed increases in T2 relaxation times in the medial compartment likely reflect the increased water content and altered collagen organization due to disease progression [1]. It is unclear why T2 values in the lateral compartment decreased during disease development. One hypothesis could be that this compartment undergoes increased cell proliferation and collagen production, which in turn could decrease T2 values, as previously seen in animal models of OA [29]. Further histological studies are needed to confirm these postulations. The contrasting changes in T2 values between the compartments were made more apparent in the medial-lateral ratios. Medial-lateral ratios are typically used in studies of cartilage morphology; including these measures in cartilage relaxometry analyses can bring insights into the relationships between joint loading and cartilage properties in OA [30].

In contrast to the study hypotheses, no increase in cartilage thickness was found in either the load-bearing VOIs of the medial compartment in early OA nor in the non-load-bearing VOIs of the medial and lateral compartments between OA radiographic severities. This differs from previous evidence of cartilage thickening in the medial load-bearing VOIs in the early stages of OA and in areas of lesser loading throughout the disease [11,12,13,14,28,31,32]. The causes for medial cartilage thickness increases observed in the early stages of the disease in the general medial knee OA population are not entirely understood [13,29]. In a healthy knee joint, anabolic and catabolic processes of cartilage are balanced to maintain tissue metabolic homeostasis [30]. Using evidence from animal models, it is suggested that early cartilage thickening may be due to a reparative, anabolic response to the initial cartilage damage, resulting in increased proteoglycan production and apparent cartilage hypertrophy [24,31,32]. In the later stages of the disease, the same anabolic signaling may exist, but its effect is likely only visible in areas of cartilage not exposed to load-induced degradation, such as the most posterior aspect of the medial condyle [26]. The lack of cartilage thickening in any ROI of the tested knees suggests that the cartilage metabolic balance in fast-progressing knees is dominated by catabolic activities. Further histological studies would be necessary to confirm this possible explanation, and comparisons of the biological processes involved in knees of various OA subtypes could reveal new therapeutic pathways in OA.

The results must be interpreted with consideration for the study limitations. A population of fast-progressing knees was chosen to provide a study group of similar progression speeds within the OAI database. This resulted in a study group with a mean BMI classified as obese. Therefore, further investigations will be necessary to assess the generalizability of the presented findings to non-obese patients and knees with slower disease progression. Furthermore, future work should consider extending the utilized method to analyzing T2 changes across various OA severities in the cartilage of the patellofemoral joint and in the tibial cartilage, both of which are also affected by disease progression [2,33]. Longitudinal cartilage changes in a control population of KL0 knees were not employed here for comparison, as these changes have been previously evaluated in the same cohort of patients as in this study [34,35]. Specifically, Buck et al., reported annualized cartilage thickness changes in healthy individuals of <0.7%/year [34]; significant changes found in this study ranged from −8.8%/year to −1.7%/year. Similarly, T2 value changes in the medial femoral cartilage of healthy individuals have been reported at 1.4%/year [35], as compared to the 4.4%/year increase found in this study. Finally, the “magic angle effect” may have influenced the T2 values in locations of cartilage oriented at about 55° relative to B_0_ [36]. However, due to the longitudinal, intra-subject design of this study along with the standardized imaging protocol, this effect likely acted as a constant additive bias to the T2 estimates in consistent VOIs; its effect was thus minimized when analyzing the differences between severity timepoints.

## 5. Conclusions

In conclusion, this study characterized cartilage T2 and thickness changes with respect to radiographic structural disease progression in a group of fast-progressing medial OA knees using an intra-subject longitudinal study design. Unique to this study was the use of VOIs spanning the entire condylar surfaces, the simultaneous analysis of relaxometry and morphology data, the application of a standardized methodology, and the longitudinal analysis of cartilage through multiple radiographic stages of OA. The results highlighted that cartilage T2 and thickness changes vary spatially over the femoral condyles. Additionally, all T2 changes occurred in the early radiographic stages of OA, whereas thickness changes occurred primarily in the later stages. These data therefore support the use of T2 relaxation time analyses in methods of detecting, potentially more sensitively than with radiography, disease-related change during early OA, a valuable period for therapeutic interventions.

## Data Availability

The source data are publicly available at https://oai.nih.gov.

## Appendix A

**Table A1:** Osteoarthritis Initiative dataset versions used for study cohort identification and analysis.

Data Collected	Dataset Name	Version Number
Radiological joint space narrowing (JSN) and Kellgren-Lawrence grade (KL)	kxr_sq_bu00	0.8
	kxr_sq_bu01	1.8
	kxr_sq_bu03	3.7
	kxr_sq_bu05	5.7
	kxr_sq_bu06	6.5
	kxr_sq_bu08	8.2
	kxr_sq_bu10	10.2
MRI quality control	mri00	0.2.3
	mri01	1.2.2
	mri03	3.2.2
	mri05	5.2.2
	mri06	6.2.2
	mri08	8.2.2
	mri10	10.2.2
Body Mass Index (BMI)	physexam00	0.2.2
	physexam01	1.2.1
	physexam03	3.2.1
	physexam05	5.2.1
	physexam06	6.2.1
	physexam08	8.2.1
	physexam10	10.2.1
Presence of rheumatoid arthritis/inflammatory disease	medhist00	0.2.2
Age at baseline	allclinical00	0.2.3
Sex	enrollees	25
